# Racial Differences in Survival among Hemodialysis Patients after Coronary Artery Bypass Grafting

**DOI:** 10.3390/ijerph10094175

**Published:** 2013-09-06

**Authors:** Jimmy T. Efird, Wesley T. O’Neal, Paul Bolin, Stephen W. Davies, Jason B. O’Neal, Curtis A. Anderson, T. Bruce Ferguson, W. Randolph Chitwood, Alan P. Kypson

**Affiliations:** 1 Department of Cardiovascular Sciences, Brody School of Medicine, East Carolina Heart Institute, East Carolina University, Greenville, NC 27834, USA; E-Mails: andersoncu@ecu.edu (C.A.A.); fergusont@ecu.edu (T.B.F.); chitwoodw@ecu.edu (W.R.C.); kypsona@ecu.edu (A.P.K.); 2 Center for Health Disparities, Brody School of Medicine, East Carolina University, Greenville, NC 27834, USA; 3 Department of Internal Medicine, Wake Forest University School of Medicine, Winston-Salem, NC 27157, USA; E-Mail: wesoneal85@gmail.com; 4 Department of Internal of Medicine, Division of Nephrology and Hypertension, Brody School of Medicine, East Carolina University, Greenville, NC 27834, USA; E-Mail: bolinp@ecu.edu; 5 Department of General Surgery, University of Virginia School of Medicine, Charlottesville, VA 22908, USA; E-Mail: sd2wf@virginia.edu; 6 Department of Anesthesia, Critical Care, and Pain Medicine, Beth Israel Deaconess Medical Center, Harvard Medical School, Boston, MA 02215, USA; E-Mail: jboneal@bidmc.harvard.edu

**Keywords:** dialysis, mortality, paradox, disparities, heart disease

## Abstract

The aim of this study was to examine racial differences in long-term survival among hemodialysis patients after coronary artery bypass grafting (CABG). To our knowledge this has not been previously addressed in the literature. Black and white hemodialysis patients undergoing first-time, isolated CABG procedures between 1992 and 2011 were compared. Survival probabilities were computed using the Kaplan-Meier product-limit method and stratified by race. Hazard ratios (HR) and 95% confidence intervals (CI) were computed using a Cox regression model. A total of 207 (2%) patients were on hemodialysis at the time of CABG. White (n = 80) hemodialysis patients had significantly decreased 5-year survival compared with black (n = 127) patients (adjusted HR = 1.9, 95% CI = 1.2–2.8). Our finding provides useful outcome information for surgeons, primary care providers, and their patients.

## 1. Introduction

Chronic kidney disease (CKD) and the progression to end-stage renal disease (ESRD) are associated with increased cardiovascular-related mortality [1]. Hemodialysis (HD) patients have an increased prevalence of risk factors for cardiovascular disease and are more likely to have multi-vessel coronary artery disease (CAD) [2]. HD patients also have been identified as a high-risk surgical group due to their decreased long-term survival after cardiac surgery [3,4,5].

Survival paradoxes are well documented among cardiovascular and HD patients and “describe the association of certain risk factors with negative outcomes in the general population and the opposite effect in certain subpopulations and *vice versa*” [6]. Conventional cardiovascular risk factors such as black race, hypercholesterolemia, hypertension, and obesity are associated with increased survival among HD dialysis [7]. The rationale for the current study was to determine if similar reverse epidemiologic findings are observed among black HD patients undergoing isolated CABG in our rural, racially dichotomous population.

## 2. Experimental Section

Details of the study database have been previously described and are summarized below [8,9,10].

### 2.1. Study Design

This was a retrospective cohort study of 207 HD patients undergoing first-time, isolated CABG at the East Carolina Heart Institute between 1992 and 2011. Demographic data, comorbid conditions, CAD severity, and surgical data were collected at the time of surgery. Only black and white patients were included to minimize the potential for residual confounding (~1% other races). Racial identity was self-reported. HD patients who died during the in-hospital postoperative period were excluded to limit the influence of short-term survival on long-term outcomes. Of these, 10 were black (mean age = 65 ± 12, 50% male) and 8 were white (mean age = 65 ± 10, 63% male). The study was approved by the Institutional Review Board at the Brody School of Medicine, East Carolina University. 

### 2.2. Definitions

HD was defined as patients with ESRD requiring dialysis preoperatively. Non-elective procedures included urgent, emergent, and salvage operations. Long-term mortality was defined as any cause of death occurring postoperatively. CAD severity was defined by the number of diseased vessels (1, 2, or 3) with at least 50% stenosis and confirmed by angiography before surgery. 

### 2.3. Setting

The East Carolina Heart Institute is a 120-bed cardiovascular hospital located in the center of eastern North Carolina, a rural region with a large black population. The institute is the largest stand-alone, tertiary referral hospital focusing on cardiovascular care in the state of North Carolina. Cardiovascular disease is the number one cause of death in North Carolina with an unequal burden occurring in eastern North Carolina [11]. The institute is a population-based tertiary referral center. Nearly all patients treated at the East Carolina Heart Institute live and remain within a 150-mile radius of the medical center.

### 2.4. Data Collection and Follow-up

The primary sources of data extraction were the Society of Thoracic Surgeons (STS) Adult Cardiac Surgery Database and the electronic medical record at the Brody School of Medicine. Cardiovascular surgery information at our facility has been reported to the STS since 1989. Data quality and cross-field validation are routinely performed by the Epidemiology and Outcomes Research Unit at the East Carolina Heart Institute. An electronic medical record was introduced at the Brody School of Medicine in 1997. Records from 1989 to 1997 were retrospectively scanned into the electronic medical record. Local and regional clinics were consolidated under a single electronic medical record in 2005 which allowed for efficient patient follow-up. The electronic medical record system applies multiple logic comparisons to reliably reduce mismatching of patient data across clinics and follow-up visits. The STS database is linked to the electronic medical record through a unique patient medical record number. 

Given the relatively stable patient catchment area for the East Carolina Heart Institute, most deaths are determined through the regional medical center electronic medical record system, follow-up letters/phone calls (primary care providers, patients), death certificates from county registrars, and/or obituary notices. Approximately 10% of deaths were exclusively determined by linkage with the National Death Index. The National Death Index also was used to validate death information captured in our electronic medical record. Linkage with the National Death Index was based on a multiple criteria, deterministic matching algorithm [12]. In our database, less than 5% of validated deaths failed to correctly match with the National Death Index.

### 2.5. Statistical Analysis

Categorical variables were reported as frequency and percentage while continuous variables were reported as mean ± standard deviation, median, and range. Variables not previously categorized were divided into quartiles prior to statistical analysis. Quartile categorization is advantageous because it limits the influence of outliers and allows for the assessment of trend across categories. Follow-up time was measured from the date of surgery to the date of death or censoring. Survival probabilities were computed using the Kaplan-Meier product-limit method. The log-rank test was used to compare survival between black and white patients. Cox proportional hazard regression models were used to compute hazard ratios (HR) and 95% confidence intervals (CI) for long-term mortality. The initial multivariable models included variables that have been previously reported to be associated with cardiovascular-related mortality, regardless of their statistical significance in our dataset. These included age, sex, hypertension, CAD severity, heart failure, and prior stroke [8]. The post-hoc addition of other variables into the model was performed in a pairwise fashion. The test statistic of Grambsch and Therneau was used to check the proportional hazards assumption [13]. Survival was truncated at 5 years due to the sparseness of events beyond this time point. Statistical significance for categorical variables was tested using the Fisher’s Exact Test and the Deuchler-Wilcoxon Procedure for continuous variables. P_Trend_ for decreasing or increasing HRs across levels of continuous variables was computed using a likelihood ratio test. There were no missing values in this study. SAS Version 9.3 (Cary, NC, USA) was used for this analysis. Statistical significance was defined as *p* < 0.05.

## 3. Results and Discussion

### 3.1. Results

A total of 13,453 patients underwent isolated CABG between 1992 and 2011. Of these, 207 (2%) patients were on HD at the time of surgery (Table 1). There were 127 (61%) black and 80 (39%) white HD patients (Table 1). A greater percentage of black patients (49%) were female than white patients (34%) (*p* < 0.05). Median follow-up for HD patients was 2.5 years. A greater percentage of white than black HD patients (43% *vs*. 28%) were on preoperative anti-platelet therapy (Table 2). No statistically significant differences were observed between black and white HD patients for postoperative complications (Table 3).

**Table 1:** Patient characteristics (N = 207).

Characteristic	Black		White		UnivariableHR (95% CI)
	n (%)	1, 3, 5 Year Survival	n (%)	1, 3, 5 Year Survival	
Overall	127 (61)	85, 62, 44	80 (39)	78, 48, 30	1.6 (1.1–2.3) ^‡^
*Age* (*Years*)					
Q1 (≤53)	38 (30)	92, 74, 67	21 (26)	61, 25, 20	1.0 Referent
Q2 (>53–60)	35 (28)	88, 66, 30	14 (18)	100, 85, 53	1.04 (0.60–1.8)
Q3 (>60–66)	25 (20)	83, 68, 54	23 (29)	87, 51, 32	1.1 (0.64–2.0)
Q4 (>66)	29 (23)	72, 39, 20	22 (28)	73, 44, 23	1.9 (1.2–3.2)
Mean ± SD	59 ± 9.7		60 ± 9.2		P_Trend_ = 0.012
Median (Range)	60 (35–80)		62 (39–77)		
*Sex*					
Male	65 (51)	92, 72, 54	53 (66)	81, 53, 35	1.0 Referent
Female	62 (49)	77, 52, 33	27 (34) *	73, 38, 21	1.6 (1.1–2.3)
*BMI* (*kg/m^2^*)					
Obese (≥30)	50 (39)	83, 67, 41	23 (29)	87, 75, 58	1.0 Referent
Overweight (25–29.9)	48 (38)	89, 63, 46	32 (40)	81, 47, 30	1.3 (0.81–2.1)
Normal (18.5–24.9)	27 (21)	80, 53, 44	22 (28)	73, 27, 5	1.9 (1.2–3.1)
Underweight (<18.5)	2 (2)	50, 50 ^†^	3 (4)	33, 33, 33	2.1 (0.64–6.9)
Mean ± SD	29 ± 5.8		27 ± 5.2		P_Trend _= 0.0091
Median (Range)	28 (18–49)		27 (17–41)		
*CAD Severity*					
1 Vessel	8 (6)	100, 73, 55	2 (3)	100, 100, 100	1.0 Referent
2 Vessel	37 (29)	86, 61, 44	27 (34)	77, 61, 48	2.0 (0.61–6.5)
3 Vessel	82 (65)	82, 61, 43	51 (64)	78, 38, 16	2.6 (0.83–8.4)
					P_Trend_ = 0.035
*Non-Elective*					
No	58 (46)	89, 64, 49	33 (41)	81, 63, 51	1.0 Referent
Yes	69 (54)	81, 61, 40	47 (59)	76, 38, 16	1.6 (1.1–2.4)
*Hypertension*					
No	11 (9)	73, 45, 27	7 (9)	83, 33, 17	1.0 Referent
Yes	116 (91)	86, 64, 46	73 (91)	78, 49, 31	0.61 (0.34–1.1)
*Diabetes*					
No	54 (43)	88, 60, 45	27 (34)	78, 44, 28	1.0 Referent
Yes	73 (57)	82, 63, 42	53 (66)	79, 50, 31	1.0 (0.71–1.5)
*Heart Failure*					
No	72 (57)	93, 69, 49	46 (58)	82, 51, 32	1.0 Referent
Yes	55 (43)	73, 53, 37	34 (42)	73, 44, 27	1.5 (1.1–2.3)
*Prior Stroke*					
No	105 (83)	87, 64, 49	71 (89)	81, 51, 33	1.0 Referent
Yes	22 (17)	71, 51, 19	9 (11)	53, 20 ^†^	2.1 (1.3-3.4)
*Previous MI*					
No	71 (56)	86, 65, 49	42 (53)	88, 47, 30	1.0 Referent
Yes	56 (44)	83, 58, 36	38 (47)	68, 49, 30	1.2 (0.85–1.8)

* *p* < 0.05 (comparison of black *vs*. white patients), Fisher’s Exact (Categorical Variables), Deuchler-Wilcoxon Test (Continuous Variables); ^†^ Last follow-up not reached; ^‡^ Black *vs*. White. BMI = body mass index; CAD = coronary artery disease; CI = confidence interval; HR = hazard ratio; Q1 = quartile 1; Q2 = quartile 2; Q3 = quartile 3; Q4 = quartile 4; MI = myocardial infarction; SD = standard deviation.

**Table 2:** Preoperative medications (N = 207).

Medication Medication	Black n (%)	White n (%)	*p*-value
Aspirin	73 (57)	53 (66)	0.24
Lipid Lowering Agents	55 (43)	34 (43)	1.0
Anticoagulants	30 (24)	17 (21)	0.74
Antiplatelet Agents	36 (28)	34 (43)	0.049
β-Blockers	75 (59)	50 (63)	0.66
Calcium Channel Blockers	55 (43)	33 (41)	0.89
Diuretics	17 (13)	16 (20)	0.24
ACE Inhibitors/ARBs	51 (40)	36 (45)	0.56
Digitalis	9 (7)	7 (9)	0.79
Nitrates	18 (14)	11 (14)	1.0
Inotropic Agents	1 (1)	1 (1)	1.0

ACE = angiotensin converting enzyme; ARB = angiotensin receptor blocker; CABG = coronary artery bypass grafting.

**Table 3:** Postoperative complications (N = 207).

Complication	Black n (%)	White n (%)	*p*-value
MI	0 (0)	1 (1)	0.39
Stroke	2 (2)	1 (1)	1.0
ARDS	1 (1)	1 (1)	1.0
Pneumonia	3 (2)	2 (3)	1.0
GI Event *	4 (3)	4 (5)	0.71

* Includes GI bleed, pancreatitis, cholecystitis, mesenteric ischemia, and other GI events. ARDS = acute respiratory distress syndrome; GI = gastrointestinal; MI = myocardial infarction.

**Figure 1:**
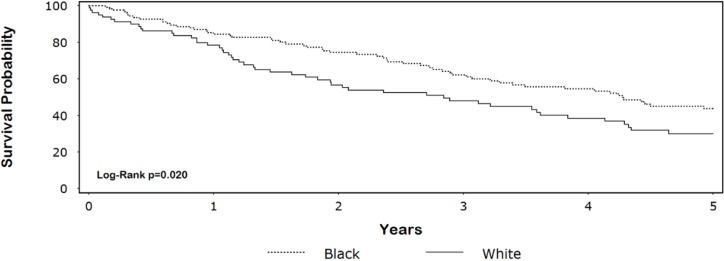
Unadjusted Kaplan-Meier 5-year survival (N = 207).

Kaplan-Meier survival curves for HD patients by race (Figure 1) were significantly different (*p* = 0.020; median survival: black = 4.3 years, white = 2.8 years). A total of 69 (50%) black patients and 30 (34%) white patients were censored. The unadjusted HR for white race was 1.6 (95% CI = 1.1–2.3) (Table 1). After adjustment for age, sex, hypertension, CAD severity, heart failure, and prior stroke, the HR was 1.9 (95% CI = 1.2–2.8). The multivariable result did not substantively change with the pairwise addition of other variables listed in Table 1 and Table 2. Approximately 87% of our patients were 50 years of age or older. Among this group, the adjusted HR remained statistically significant (HR = 1.6, 95% CI = 1.05–2.6, *p* = 0.029).

### 3.2. Discussion

Our results demonstrate a survival disadvantage for white HD patients after CABG, which is consistent with the general HD population. To the best of our knowledge, this is the first study to report this finding among HD patients receiving CABG. In contrast, a study of HD patients after heart valve replacement did not observe a survival difference by race (HR = 1.01, 95% CI = 0.95–1.09) [14]. The conflicting results may be due to demographic differences. For example, a higher percentage of patients in our study were black (39% *vs*. 31%) and above age 45 (93% *vs*. 80%) compared with the above study. Differences in inclusion/exclusion criteria, patient acuity, operative procedure, comorbid conditions, and in-hospitalization deaths also could explain the inconsistent findings between the studies.

Reverse epidemiologic findings are commonly reported in the HD literature [15,16,17,18]. For example, white HD patients are more likely to die sooner than black HD patients. We observed a similar reverse finding among HD patients following CABG, even though risk factor profiles were similar between blacks and whites for the variables presented in Table 1. This differs from the general CABG population in which whites observe a survival advantage [19,20]. Explanations for this finding may include differences in genetics, nutritional status, inflammation, and sensitivity to dialysis [17,21]. The latter factors were not included in the current study. Nonetheless we cannot rule out the possibility that the above mentioned survival paradox observed in our study reflects the observational nature of epidemiologic studies [6]. Retrospective cohort studies are excellent for the generation of hypotheses, however, they are unable to prove causality. Alternatively, the reverse epidemiologic effects may be due to residual confounding, inappropriate adjustment, or Simpson’s Paradox.

### 3.3. Strengths and Limitations

The current study is strengthened by its comparatively large sample size and long-term follow-up. Furthermore, we were able to accurately determine time of death using a combination of the National Death Index and our comprehensive electronic medical record.

The retrospective design of our study may have introduced selection bias. Although we adjusted for known clinically relevant variables, we acknowledge that other unmeasured factors could have influenced the results due to the retrospective nature of the study. The status of several variables may have changed over time. We did not adjust for these variables in a time-dependent manner due to their potential to be in the causal pathway. Postoperative complications were not included in our analysis because of their time-dependent status. Also, status of revascularization (incomplete *vs*. complete) was not included due to inconsistencies in the definition of this variable [22].

Racial identify was self-reported and this could have resulted in misclassification bias. Furthermore, race may reflect cultural identification rather than genetic identity and this distinction was beyond the scope of our study. Cause of death is not recorded in the National Death Index and patients’ mortality could have had little to do with their heart disease and also could have contributed to misclassification bias.

Higher body mass index has been associated with a lower in-hospital mortality risk among heart failure patients [23]. The number of underweight HD patients in our sample was small and we were unable to perform a similar comparison. Nonetheless, HD patients who were underweight had a greater risk of mortality than those who were obese (HR = 2.1, 95% CI = 0.64-6.9), although the confidence interval was wide and spanned unity.

Patients in this study were recruited over a relatively long period, over which patient characteristics, practice methods, and clinical care may have changed considerably. We did not account for patients who were previously on peritoneal dialysis or who were switched from HD any time after surgery. Also, the duration of dialysis and the acute *versus* chronic nature of their kidney disease was not recorded and this has been shown to influence survival [17]. We did not adjust our model for cause of CKD because the majority of patients had similar etiology (hypertension, diabetes, and obesity) [24].

HD patients with off-pump CABG have been shown to have better survival compared with on-pump procedures [5]. No distinction was made between these operations and long-term survival after CABG due to the small number of off-pump cases (n = 25). The results also are from a single center that may not generalize to the overall population.

Recently, it has been suggested that the commonly cited increased mortality risk observed for white HD patients applies only to older adults [16]. Less than 15% of the 207 HD patients in our study were under the age of 50 and consequently we were limited in our ability to directly address this question. However, among elderly patients in our sample we observed a slight decrease in effect size (white *vs*. black, adjusted HR = 1.6) compared with the overall sample (white *vs*. black, adjusted HR = 1.9), suggesting that the increased risk of mortality for white *vs.* black patients also holds among younger patients. This is supported by a recent study, which reported that the survival advantage of black patients holds for age groups above 30 years of age [25]. However, we cannot rule out potential effect modification by age in our study due to our limited sample size.

Our use of quartile boundaries, while desirable for minimizing the influence of outliers, may have yielded overly broad categories and the potential for residual confounding. However, the substitution of continuous variables in our models did not materially alter results. Additionally, multivariable Cox regression models, rather than propensity score matching, were used to control for confounding because of potential “non-collapsibility bias” inherent to logistic regression-based propensity scores [26].

Data regarding socioeconomic position, education, and income also were not collected in our study and these factors may have influenced survival [27]. For example, factors related to socioeconomic position, historic discrimination, and related comorbidities could have influenced which patients were selected to receive surgery. Twenty-eight (97%) of the 29 counties in eastern North Carolina fall below the national per capita income of $27,915, with half reporting a value less than $20,000 [28]. Similarly, 90% of the counties have a higher percentage of blacks than the national value of 13.1% [28]. Selection bias also could have been due to related socioeconomic factors such as lack of insurance coverage, transportation, and overall poor health status. We were unable to reliably estimate socioeconomic position using zip codes because a large percentage of patients in our region live in rural areas with postal box addresses. 

## 4. Conclusions

White HD patients were observed to have worse survival following CABG than black HD patients. This effect is paradoxically reversed from the general non-HD population. The findings of this study have important implications for identifying at-risk HD patients who may require early preoperative intervention and close postsurgical follow-up. Further studies with larger sample sizes are needed to confirm our findings and to explore the underlying causes of this potential racial difference in survival among HD patients.
